# Engineered Nanomaterials, Microbial Community Responses, and Fe-Mediated Regulation of As and Cd Fate in the Flooded Rice Rhizosphere: A Mechanistic Synthesis

**DOI:** 10.3390/microorganisms14061336

**Published:** 2026-06-14

**Authors:** Yinghui Gu, Yimeng Ren, Xiaodan Wang, Kai Song, Lihui Zhang

**Affiliations:** 1 School of Life Science, Changchun Normal University, Changchun 130032, China; qx202510003@stu.ccsfu.edu.cn (Y.G.); rym351528@163.com (Y.R.); qx202510031@stu.ccsfu.edu.cn (X.W.); 2 Institute of Innovation Science and Technology, Changchun Normal University, Changchun 130032, China

**Keywords:** rice rhizosphere, sediment-water microinterface, engineered nanomaterials, bacterial community, iron plaque, biogeochemical cycling, cross-interface migration

## Abstract

The flooded rice rhizosphere is a continuous reactive interface composed of sediment, porewater, root-surface oxic microdomains, and iron plaque, where redox processes and Fe cycling regulate Cd/As speciation, bioavailability, and plant accumulation. Engineered nanomaterials (ENMs) have shown potential for reducing Cd/As uptake in rice, but the coupled roles of microbial community responses, iron-plaque gating, and cross-interface elemental migration remain insufficiently integrated. This review synthesizes the current evidence on ENM transformation and partitioning at flooded rhizosphere microinterfaces, focusing on front-end speciation changes, root-surface retention, microbial functional regulation, and plant sequestration or transport. Correlative evidence suggests that rhizosphere microorganisms are associated with altered redox conditions, Fe cycling, As methylation potential, and metabolite secretion, which may influence Cd/As partitioning and cross-interface migration. However, direct causal validation of the complete ENM transformation–microbial response–Fe cycling–Cd/As flux–grain accumulation sequence within a single integrated system remains lacking. We further discuss how elevated CO_2_, micro-/nanoplastics, Fe/DOM dynamics, and water management regimes may modify this framework, and we identify Sb as a theoretical boundary case because direct ENM–rice evidence remains limited. Finally, we highlight the need to integrate spatial tracing and imaging methods, including persistent luminescence tracing, LA-ICP-MS, NanoSIMS, and µ-XRF/µ-XANES, with metaomics to connect particle localization, microbial function, and contaminant fate.

## 1. Introduction

Rice is a globally important staple crop. Under prolonged flooded cultivation, its rhizosphere develops into a continuous reactive system composed of sediments, porewater, root-surface oxic microdomains, and iron plaque. In this sediment, reprecipitation cycling of Fe/Mn minerals occurs, thereby influencing the speciation, mobility, and bioavailability of contaminant elements such as Cd and As [1,2,3,4,5,6]. Particle-size sieving has been applied to remediate Pb/Cu-contaminated agricultural soils, indicating that heavy metal contamination is closely associated with soil particle-size fractions and interfacial partitioning processes [7]. Therefore, the mitigation of metal/metalloid contamination in paddy fields should not be simplified as a single chemical immobilization problem. Rather, it should be understood as a multi-interface regulatory process governed by reducing conditions, iron-plaque dynamics, and microbial ecological interactions.

In recent years, ENMs have been used to reduce Cd/As accumulation in rice and have shown efficacy across different material systems [8,9,10]. Several influential reviews have examined ENMs in agricultural remediation contexts [11,12,13], the response of rhizosphere microbiomes to nanoparticle exposure [14,15], and the role of iron plaque in controlling As/Cd uptake by rice [16,17]. However, these bodies of work share a common limitation: they treat physicochemical immobilization, microbial responses, and iron-plaque dynamics as largely separate topics, and few explicitly address the flooded, reducing rhizosphere as a continuous reactive system in which these processes are mechanistically coupled. Moreover, existing reviews rarely distinguish between taxonomic community shifts and functional rewiring at the level of Fe cycling, As methylation, and metabolite secretion, nor do they integrate spatial tracing evidence to evaluate particle fate across the rhizosphere–iron plaque–tissue continuum. In addition, current studies often rely primarily on endpoint evidence, such as decreased grain concentrations or improved antioxidant indices, and attribute mitigation effects to direct physicochemical processes, including adsorption, precipitation, or ionic antagonism. In practice, once ENMs enter the flooded rhizosphere, they are jointly regulated by reducing conditions, dissolved organic matter (DOM), and microbial metabolic networks, leading to surface reconstruction, dissolution-transformation, and interfacial redistribution. The resulting microbial ecological responses are not merely the secondary phenomena associated with plant contamination responses. They may represent a potential intermediary layer linking interfacial processes with endpoint accumulation. However, the causal strength of this linkage remains insufficiently resolved because most available evidence is based on community composition, bulk fractionation, or endpoint measurements rather than direct microbial functional validation. Considering that combined Cd/As contamination is common in Asian rice-growing regions [18,19], this review focuses primarily on Cd and As. Because direct evidence for Sb in ENM-mediated rice mitigation systems remains limited, Sb is considered only as a boundary reference for mechanistic extrapolation.

The present review addresses these gaps by proposing an integrated analytical framework—“nanomaterial perturbation–microbial mediation–interfacial processes–plant responses”—that explicitly treats the flooded rice rhizosphere as a spatially continuous system and places microbial functional regulation, rather than compositional change, at the center of the mechanistic chain. This framework differs from prior work in three specific respects: (1) it integrates ENM surface reconstruction under reducing conditions with downstream microbial functional responses within a single conceptual model; (2) it treats iron plaque as a dynamic, microbially regulated gating interface rather than a static physical barrier; and (3) it identifies spatial omics and persistent luminescence tracing as methodological priorities for advancing from correlative inference to causal validation. Two interrelated questions require further attention in this field: through which continuous rhizospheric processes do ENMs influence Cd/As accumulation, and can these processes be supported by direct spatial and functional evidence? Conventional endpoint measurements cannot resolve the microregional fate of particles across the rhizosphere, iron plaque, and plant tissues. Persistent luminescent and long-afterglow nanoplatforms can reduce interference from plant tissue autofluorescence and improve the spatiotemporal resolution of particle cross-interface migration [20,21]. These methods provide a methodological basis for advancing the mechanistic interpretation from a correlative description toward spatially verifiable process models.

**Figure 1:**
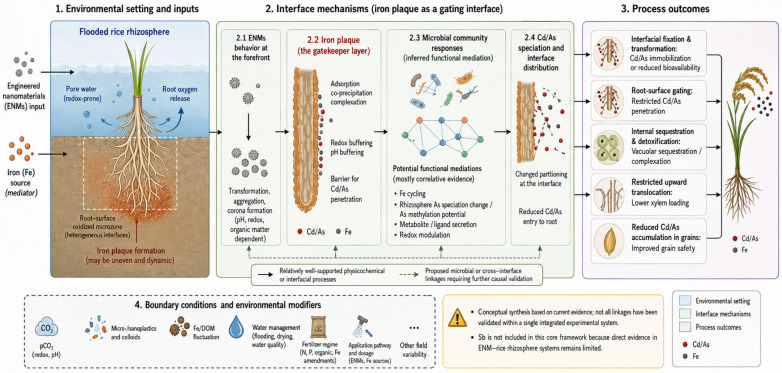
Conceptual framework linking ENM transformation, microbial community responses, Fe-mediated interfacial regulation, and Cd/As fate in the flooded rice rhizosphere. Solid arrows indicate relatively well-supported physicochemical or interfacial processes, whereas dashed arrows indicate proposed microbial or cross-interface linkages requiring causal validation. Sb is not included because direct evidence for Sb regulation in ENM–rice rhizosphere systems remains limited.

Based on these questions, this review focuses on the flooded rice rhizosphere microinterface and proposes an analytical framework of “ENM perturbation-microbial mediation-interfacial processes-plant responses” (Figure 1). Several figures in this revieware conceptual/schematic frameworks intended to visually synthesize the mechanistic relationships discussed in the text. As is standard in review papers, these figures integrate evidence from multiple independently published studies and are not representations of a single experimental system. In all cases, the evidence supporting each mechanistic node is evaluated explicitly in the text and in the corresponding summary table, with distinctions drawn between experimentally validated mechanisms, correlative observations, and hypothetical extrapolations. The review discusses (1) environmental transformation and interfacial partitioning of ENMs within continuous microinterfaces of the flooded rhizosphere; (2) the role of rhizosphere bacterial community reassembly and functional changes in regulating elemental fate; (3) a continuous mechanistic chain comprising rhizospheric immobilization, iron-plaque gating, internal sequestration, and restricted long-distance transport; (4) boundary constraints imposed on this mechanistic chain by complex scenarios, including elevated CO_2_ and micro-/nanoplastic coexistence; and (5) the application prospects of spatial tracing, multi-omics, and causal manipulation for mechanistic validation.

## 2. Environmental Behavior of ENMs and the Background of Elemental Transformation at the Sediment–Water Microinterface of the Flooded Rice Rhizosphere

**Figure 2:**
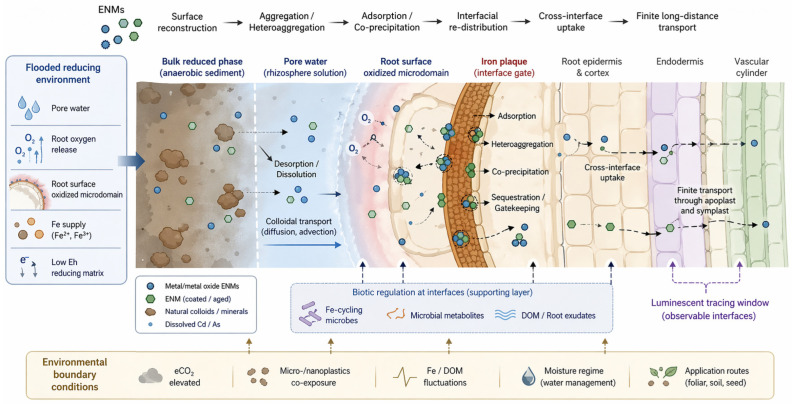
Schematic representation of the environmental behavior, interfacial fate, and cross-interface migration of ENMs within continuous microinterfaces of the flooded rice rhizosphere. This conceptual synthesis summarizes proposed ENM transformation, interfacial retention, and restricted tissue migration processes across the rhizosphere–iron plaque–plant continuum. Solid arrows indicate evidence-supported processes, whereas dashed or schematic pathways indicate hypothesized linkages requiring further spatial validation.

After ENMs enter the flooded rice rhizosphere, their primary effects involve spatial distribution and chemical speciation across the bulk reducing phase, porewater, root-surface oxic microdomains, and iron-plaque interfaces, rather than the plant endpoint phenotypes themselves. Redox fluctuations, Fe cycling, DOM, and rhizosphere metabolic networks can jointly drive ENM surface reconstruction and interfacial redistribution. Accordingly, the rhizospheric behavior of ENMs should be regarded as a continuous process composed of environmental transformation, interfacial gating, and tissue migration, which provides the material basis for microbial functional responses and Fe-As-Cd biogeochemical transformations (Figure 2).

### 2.1. Surface Reconstruction and Front-End Transformation of ENMs Under Flooded Reducing Conditions

**Figure 3:**
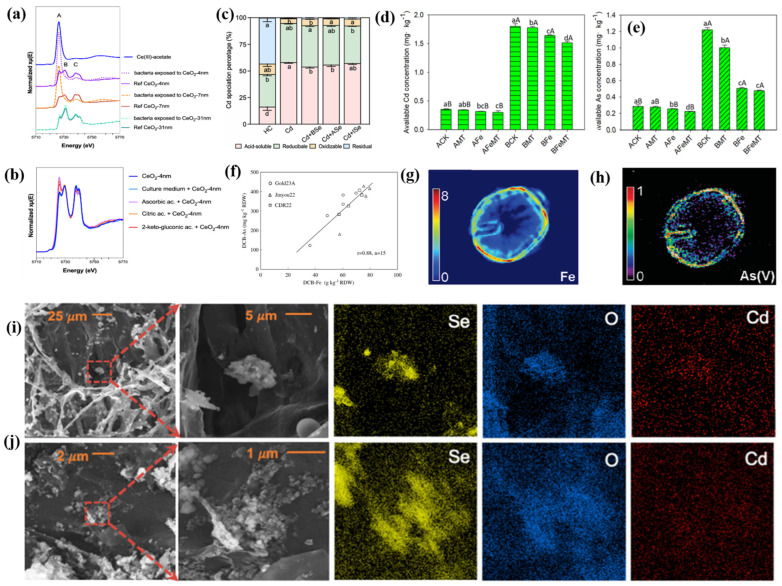
ENM transformation, contaminant immobilization, and iron-plaque retention at flooded rice rhizosphere interfaces. (**a**,**b**) Bacterially and metabolite-mediated surface transformation of CeO_2_ NPs, illustrating nanoscale redox reconstruction at the front-end rhizosphere interface. (**c**) Redistribution of Cd among operationally defined soil fractions after Se NP treatment, indicating reduced Cd bioavailability and enhanced stabilization. (**d**,**e**) Effects of nZVI combined with melatonin on soil-available Cd and As, respectively, showing ENM-associated reductions in contaminant bioavailability. (**f**) Coupled enrichment of Fe and As in DCB-extractable iron plaque, illustrating Fe-mediated As retention at the root surface. (**g**,**h**) Spatial distributions of Fe and As(V), respectively, showing Fe-rich plaque domains and associated As(V) retention at the root surface. (**i**,**j**) Colocalization of Se, O, and Cd in the root-surface region under Se NP treatment, indicating ENM-associated interfacial retention of Cd. Notes: ENMs, engineered nanomaterials; NPs, nanoparticles; CeO_2_ NPs, cerium oxide nanoparticles; Se NPs, selenium nanoparticles; nZVI, nano zero-valent iron; DCB, dithionite–citrate–bicarbonate-extractable fraction; In subgraphs c, d, and e, different letters indicate statistically significant differences among groups (*p* < 0.05). Panels (**a**,**b**) are reprinted with permission from Ref. [22]. Copyright 2022 American Chemical Society. Panel (**c**) and panels (**i**,**j**) are reprinted with permission from Ref. [23]. Copyright 2024 American Chemical Society. Panels (**d**,**e**) are reprinted with permission from Ref. [24]. Panel (**f**) is reprinted with permission from Ref. [3]. Copyright 2004 Society for Experimental Biology. Panels (**g**,**h**) are reprinted with permission from Ref. [4]. Copyright 2010 American Chemical Society.

Flooding in paddy fields generates low redox potential and promotes dissolution-reprecipitation cycling of reactive components such as Fe(III) minerals and organic matter. Under these reducing conditions, the surface coordination, charge state, and particle morphology of metallic or metal-oxide ENMs may be reconstructed, and some materials may release ions. Taking CeO_2_ NPs as an example, particles of different sizes exhibit size-dependent Ce(IV) to Ce(III) reduction under the influence of rhizosphere bacteria, indicating that particle size is an important factor controlling rhizospheric biotransformation activity [22] (Figure 3a). Low-molecular-weight rhizosphere metabolites, such as 2-ketogluconic acid and citric acid, can also induce surface reduction of smaller CeO_2_ NPs, suggesting that microbial metabolites may directly participate in the redox reconstruction of ENMs [22] (Figure 3b).

In contaminated rice systems, these processes can further alter Cd/As bioavailability. Biosynthesized Se NPs decreased the proportion of acid-extractable Cd in soil and promoted its transformation into the more stable oxidizable fraction [23] (Figure 3c). The combined treatment of nZVI and melatonin altered the distribution of soil-available Cd/As and dithionite–citrate–bicarbonate (DCB)-extractable Fe, Cd, and As, suggesting a possible coupling between Fe-phase reconstruction and the regulation of Cd/As bioavailability [24] (Figure 3d,e). Overall, ENMs can reshape the front-end chemical environment of the flooded rhizosphere through size-dependent valence transformation, metabolite-induced reduction, adsorption–complexation, and Fe-phase coupling, thereby altering the electron-acceptor/donor landscape available to rhizosphere bacteria.

### 2.2. Root-Surface Iron Plaque: A Dynamic Gating Interface for Particles and Contaminant Elements

Radial oxygen loss from rice roots can induce local Fe^2+^ oxidation and deposition at the root surface, forming Fe(III)-rich iron plaque. This process is typically accompanied by gradients in rhizosphere Eh, pH, and Fe speciation, which are important preconditions for iron-plaque formation [25]. Root-surface iron plaque can retain As through Fe-associated adsorption or coprecipitation, as shown by the coupled enrichment of DCB-extractable Fe and As and by spatial Fe/As(V) colocalization at the root surface [3,4] (Figure 3f–h). Therefore, iron plaque should not be regarded as a homogeneous static barrier, but rather as a dynamic gating structure regulating contaminant-element flux across the root-surface interface.

Iron plaque can retain ENM particles and Cd/As through surface adsorption, heteroaggregation, and coprecipitation. In rice roots treated with Se NPs, high-resolution elemental imaging revealed the colocalization of Cd, Se, and O in the root-surface region, suggesting that Se NPs may enhance Cd retention at the root-surface interface through adsorption, complexation, or coprecipitation [23] (Figure 3i,j). The synchronous changes in DCB-Fe, DCB-Cd, and DCB-As induced by nZVI treatment further support the key screening role of the iron-plaque interface from the perspective of elemental pool partitioning [24]. Importantly, iron-plaque formation and dissolution are also influenced by Fe-reducing/Fe-oxidizing microbial communities and rhizosphere metabolites. Therefore, its gating effect is shaped jointly by interfacial chemistry and microbial ecology [26].

### 2.3. Regulatory Networks of DOM and Microbial Metabolites in Interfacial Partitioning

**Figure 4:**
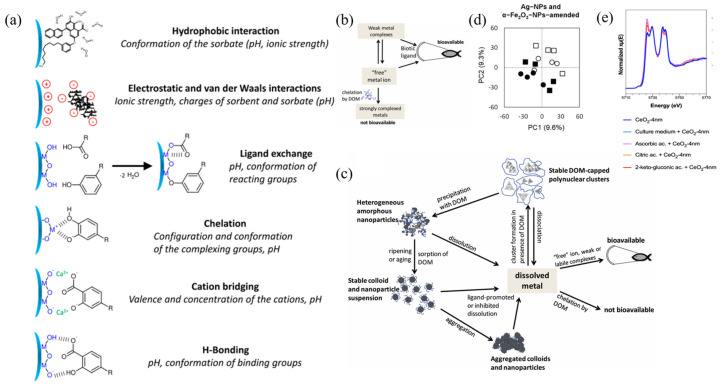
Mechanistic framework of DOM- and microbial metabolite-mediated regulation of ENM–Fe mineral–contaminant behavior at the root-surface interface. (**a**) Major interaction mechanisms between DOM and colloid/nanoscale-particle surfaces. (**b**,**c**) DOM-mediated migration and transformation processes of metals, nanoscale particles, and colloids. (**d**) Effects of particle-type ENMs and the plant rhizosphere environment on bacterial community structure. (**e**) Surface reduction of CeO_2_ NPs induced by low-molecular-weight rhizosphere metabolites. Notes: DOM, dissolved organic matter; ENMs, engineered nanomaterials; NPs, nanoparticles. (Panel (**a**) is reprinted with permission from Ref. [27]. Copyright 2014 American Chemical Society. Panels (**b**,**c**) are reprinted with permission from Ref. [29]. Copyright 2011 American Chemical Society. Panel (**d**) is reprinted from Ref. [30]. Panel (**e**) is reprinted with permission from Ref. [22]. Copyright 2022 American Chemical Society).

The retention of ENMs and contaminant elements at the iron-plaque interface is continuously regulated by DOM and microbial metabolites [27,28,29]. Humic substances and low-molecular-weight organic acids can alter particle interfacial affinity and thermodynamic fate through surface coating, site competition, and local acidification [27] (Figure 4a). DOM also affects precipitation, dissolution, aggregation, and migration among dissolved metals, nanoscale particles, and colloids, thereby linking particle interfacial behavior with contaminant bioavailability [29] (Figure 4b,c). Root exudates and bacterial metabolites not only influence microenvironmental pH, carbon-source composition, and bacterial community structure [30] (Figure 4d), but may also directly participate in interfacial redox reactions [22] (Figure 4e). Therefore, DOM and microbial metabolites constitute important chemical mediators connecting exogenous nanoscale perturbation, Fe mineral-phase changes, and Cd/As cross-interface influx. The strength of this regulation depends on organic ligand type, Fe-phase status, particle surface properties, and local microbial metabolic activity.

### 2.4. Restricted Tissue Translocation of ENMs and Spatial Methodological Validation

After front-end transformation and iron-plaque retention, only a small proportion of ENMs or their secondary transformation products may overcome multiple root-surface barriers and enter plant tissues. Because flooded soils contain complex organic matter and strong signal interference at the iron-plaque interface, conventional fluorescent tracing is susceptible to interference from endogenous plant pigments, making accurate quantification of particle microregional fate difficult. Long-afterglow or persistent luminescent nanoplatforms can continue emitting light after excitation ceases, thereby reducing background noise and providing high-signal-to-noise spatial evidence for resolving particle distribution along the “rhizosphere-iron plaque-vascular tissue” continuum [20,21]. Existing plant-system studies have shown that more than 99.45% of exogenous submicrometer particles are retained in roots, with markedly restricted translocation to aboveground tissues [20]. This finding suggests that long-term particle retention at root-surface and intraroot interfaces may represent an important source of sustained effects on rhizosphere microenvironments and microregional microbiomes.

In summary, the environmental behavior of ENMs in the flooded rice rhizosphere is a continuous screening process involving front-end surface reconstruction, iron-plaque retention, interfacial material redistribution, and restricted cross-interface migration. The introduction of spatial methods, such as luminescence tracing, enables higher-resolution visual validation of these interfacial behaviors. On this basis, the action nodes, microbial responses, and evidence maturity still differ among different types of ENMs (Table 1).

Despite the mechanistic insights described above, several important methodological limitations must be acknowledged when interpreting the existing evidence. First, the majority of studies on ENM transformation and interfacial partitioning in flooded rice systems are conducted under pot or hydroponic conditions with homogeneous soils, well-defined contaminant loadings, and short exposure durations (typically ≤ 8 weeks). These conditions may substantially overestimate the transformation rates and immobilization efficiencies achievable in heterogeneous paddy field soils with complex organic matter, competing anions, and variable redox histories. Second, many studies infer ENM-induced changes in Fe-phase dynamics or Cd/As speciation from bulk sequential extractions (e.g., BCR or DCB fractionation), which do not provide spatially resolved information and may misattribute changes in operationally defined fractions to specific mineralogical transformations. Third, the coupling between Fe-phase reconstruction and microbial community composition is frequently inferred from co-occurrence rather than demonstrated mechanistically. In most cited studies, it remains possible that the observed changes in Fe speciation are primarily driven by ENM physicochemistry rather than microbially mediated redox processes. These limitations do not invalidate the evidence but do restrict the strength of mechanistic interpretation, and they should be borne in mind when evaluating the generalizability of the proposed framework.

## 3. Bacterial Community Assembly and Biogeochemical Functional Responses at Rhizosphere Microinterfaces Under Nanoscale Perturbation

**Figure 5:**
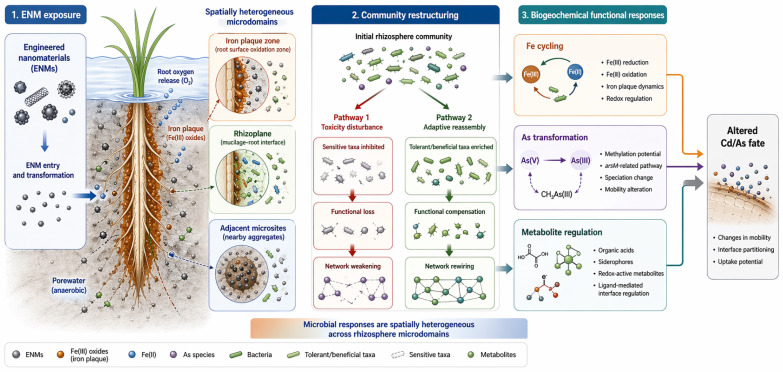
Schematic representation of rhizosphere bacterial community reshaping and biogeochemical functional responses under nanoscale perturbation in the flooded rice rhizosphere. This conceptual framework synthesizes evidence from ENM exposure and rhizosphere microbiome studies rather than a single experimental dataset. Solid arrows indicate relationships supported by experimental or sequencing-based evidence, whereas dashed arrows indicate inferred functional linkages requiring further validation.

After ENMs enter the flooded rice rhizosphere, they are usually preferentially distributed at heterogeneous interfaces, such as root-surface iron plaque, the mucilage layer, and microaggregates, thereby creating a highly spatially differentiated background for microbial exposure. Their ecological effects should, therefore, be evaluated not only by plant endpoint phenotypes but also by changes in rhizosphere bacterial community assembly, network interactions, and functional expression patterns (Figure 5). This microbial response layer is proposed here as a potential intermediary linking ENM interfacial behavior with the endpoint fate of Fe-mediated Cd/As transformation, although the causal strength of this linkage varies among studies and remains incompletely validated.

### 3.1. Community Structural Reassembly: Dual Trajectories of Toxic Perturbation and Adaptive Response

**Figure 6:**
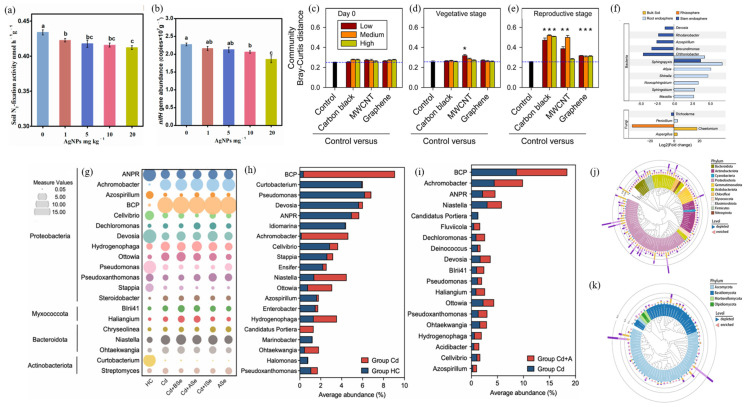
Toxic perturbation and adaptive reassembly patterns of rhizosphere microbial communities under ENM exposure. (**a**,**b**) Inhibitory effects of Ag NPs on soil nitrogen-fixation activity and nifH gene abundance. (**c**–**e**) Effects of carbon-based ENMs on soybean rhizosphere prokaryotic community composition. Error bars indicate the standard error of the mean. * indicates significant difference at *p* < 0.05 compared with the no amendment control (** *p* < 0.01; *** *p* < 0.001). (**f**) Enrichment of plant growth-promoting genera, including Sphingopyxis, Sphingobium, and Novosphingobium, after carbon nanosol treatment. (**g**–**i**) Enrichment of potential beneficial bacteria such as Azospirillum in rice roots, shoots, and rhizosphere soil under Se NP treatment. (**j**,**k**) Regulation of beneficial bacterial and fungal indicator taxa in the rhizosphere by carbon nanosol under field conditions. Notes: ENMs, engineered nanomaterials; NPs, nanoparticles; Ag NPs, silver nanoparticles; Se NPs, selenium nanoparticles; nifH, gene encoding nitrogenase reductase; Different letters in the image indicate statistically significant differences among groups (*p* < 0.05). (Panels (**a**,**b**) are reprinted from Ref. [39]. Panels (**c**–**e**) are reprinted with permission from Ref. [35]. Copyright 2018 American Chemical Society. Panel (**f**) is reprinted from Ref. [33]. Panels (**g**–**i**) are reprinted with permission from Ref. [23]. Copyright 2024 American Chemical Society. Panels (**j**,**k**) are reprinted with permission from Ref. [34]. Copyright 2024 The Authors. Published by Springer Nature).

Rhizosphere microdomains receive abundant carbon inputs and experience strong redox fluctuations, making them relatively sensitive to ENM exposure. ENM-induced shifts in community assembly can be summarized as two trajectories: toxic perturbation and adaptive reassembly. When the exposure dose or reaction activity exceeds a certain threshold, sensitive functional taxa are often the first to be suppressed. For example, high-dose Ag NPs significantly reduce soil nitrogen-fixation activity and nifH gene abundance and decrease the abundance of key taxa, such as Azospirillum and Rhizobium [39] (Figure 6a,b). This suggests that, even when the overall diversity does not change substantially, the loss of key functional taxa may alter the elemental transformation capacity at microinterfaces. Conversely, rhizosphere systems may undergo adaptive reassembly through enrichment of tolerant or plant-growth-promoting microorganisms. Under Cd stress, Se NP treatment promotes rice growth and enriches potentially beneficial taxa such as Azospirillum [23] (Figure 6g–i). Under field conditions, carbon nanosol can also regulate beneficial bacterial and fungal indicator taxa in the rhizosphere, demonstrating a potential for reshaping plant-beneficial microbial networks [33,34] (Figure 6f,j,k). It should be noted that community structural changes are not determined solely by material type; they are jointly influenced by ENM physicochemical properties, exposure dose, plant developmental stage, rhizosphere carbon sources, and soil background [30,35]. In particular, the effect of carbon-based ENMs on soybean rhizosphere prokaryotic communities exhibits a clear growth-stage dependence [35] (Figure 6c–e). Therefore, the direction of ENM effects on rhizosphere microorganisms should be interpreted in a stratified manner according to exposure intensity and ecological context.

It should also be noted that most studies summarized in this subsection rely primarily on 16S rRNA amplicon sequencing or marker-gene abundance to infer microbial responses. These approaches identify changes in relative taxonomic abundance or selected functional markers, but they do not directly demonstrate realized biogeochemical activity. Therefore, the enrichment of putative plant-beneficial or contaminant-transforming taxa should be interpreted as compositional evidence suggesting potential functional shifts, rather than as direct proof of enhanced nutrient cycling, Fe transformation, or Cd/As immobilization.

### 3.2. From Taxonomic Shifts to Functional Rewiring: Fe Cycling, As Transformation, and Metabolic Network Interactions

**Figure 7:**
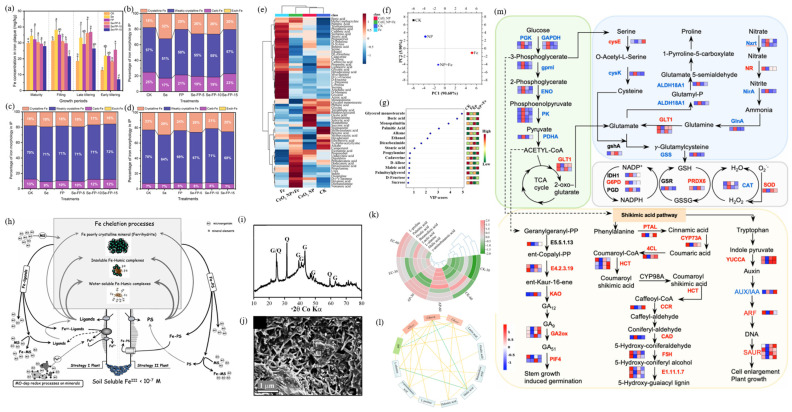
Proposed links between rhizosphere microbial functional rewiring, Fe cycling, metabolite secretion, and Cd/As interfacial transformation. (**a**–**d**) Regulation of root-surface iron-plaque formation and Fe speciation in iron plaque by Se-Fe-P. Different letters in the image indicate statistically significant differences among groups (*p* < 0.05). (**e**–**g**) Reconstruction of metabolic profiles in rice-planted soil under CeO_2_ NP and Fe^2+^ coexistence. (**h**) Microbial siderophore and plant root exudate-mediated Fe chelation and uptake processes. (**i**,**j**) Fe mineral-phase transformation induced by microbial sulfidogenesis. (**k**,**l**) Accumulation of rhizosphere metabolites, including malic acid and L-proline, is promoted by phosphate-solubilizing bacteria. (**m**) Activation of rice stress-resistance metabolic pathways, including glutathione metabolism, phenylpropanoid biosynthesis, and hormone signaling, by Se NPs. Notes: ENMs, engineered nanomaterials; NPs, nanoparticles; Se-Fe-P, selenium–iron–phosphorus combined regulatory system; CeO_2_ NPs, cerium oxide nanoparticles. (Panels (**a**–**d**) are reprinted with permission from Ref. [40]. Copyright 2024 Elsevier B.V. Panels (**e**–**g**) are reprinted with permission from Ref. [32]. Copyright 2020 American Chemical Society. Panel (**h**) is reprinted with permission from Ref. [26]. Copyright 2013 Springer-Verlag Berlin Heidelberg. Panels (**i**,**j**) are reprinted with permission from Ref. [41]. Copyright 2011 Elsevier Ltd., Oxford, United Kingdom.Panels (**k**,**l**) are reprinted with permission from Ref. [42]. Copyright 2021 Elsevier Ltd., Oxford, United Kingdom. Panel (**m**) is reprinted with permission from Ref. [23]. Copyright 2024 American Chemical Society.).

Taxonomic changes in community composition alone are insufficient to explain the altered Cd/As environmental fate, and this limitation is directly relevant to much of the evidence cited in this review. Of greater mechanistic significance would be changes at the functional levels of Fe cycling, metalloid transformation, and metabolite secretion. However, in most cited studies, such functional changes are inferred from compositional shifts, bulk fractionation patterns, or metabolite profiles rather than directly demonstrated through metatranscriptomics, enzyme activity assays, stable-isotope probing, or targeted functional-gene expression. Therefore, the mechanistic interpretations in this section should be viewed as working hypotheses supported by consistent but largely correlative evidence. In As mitigation systems, regulatory strategies such as Se-Fe-P can decrease soil-available As, promote root-surface iron-plaque formation, and alter Fe speciation within iron plaque, thereby reducing the risk of As migration to rice grains [40] (Figure 7a–d). This type of evidence suggests that exogenous regulation alters not only the apparent distribution of contaminant elements but also may influence rhizospheric As migration and transformation potential by modifying iron-plaque formation and Fe speciation within the plaque. Regarding Fe cycling, the coexistence of CeO_2_ NPs and Fe^2+^ can alter the bacterial composition and metabolic profiles in rice-planted soils, indicating that Fe-cycle-related microbial communities respond to nanoscale perturbation and may further influence microregional electron exchange and iron-plaque dissolution–deposition dynamics in the rhizosphere [32] (Figure 7e–g). However, this functional linkage remains to be directly validated under flooded rice rhizosphere conditions using metatranscriptomics, enzyme-activity assays, or stable-isotope probing. Meanwhile, microbially secreted small-molecule metabolites, such as organic acids, siderophores, and sulfur-containing metabolites, may function as chemical hubs for interfacial reactions [26,41]. Siderophores and root exudates can influence Fe availability and mobility through Fe chelation and uptake processes [26] (Figure 7h), whereas microbial sulfidogenesis may induce Fe mineral-phase transformation and further influence Fe-As coupling [41] (Figure 7i,j). Contaminant-element activation and plant defense are also associated with microbial metabolic remodeling. Phosphate-solubilizing bacteria upregulate amino-acid and carbon-metabolism pathways during enhanced Cd activation, promoting the accumulation of rhizosphere metabolites such as malic acid and L-proline [42] (Figure 7k,l). In rice systems, Se NPs can activate stress resistance metabolic pathways, including glutathione metabolism and phenylpropanoid biosynthesis, suggesting potential coordination between microbial metabolic changes and plant biochemical defense [23] (Figure 7m). Therefore, the effects of ENMs and related regulatory strategies on rhizosphere microorganisms should be validated beyond “taxonomic shifts” and at the levels of “functional expression and metabolic flux”.

### 3.3. Spatially Heterogeneous Responses of Rhizosphere Microdomains and Frontiers in Spatial Omics Validation

Most existing evidence for microbial responses is derived from sequencing of bulk soil or homogenized rhizosphere soil, which can obscure spatial heterogeneity among root-surface iron plaque, the epidermal mucilage layer, and peripheral aggregates. Root-surface iron plaque is enriched in Fe speciation transformations and organic ligands and may represent a microecological hotspot that responds first to nanoscale perturbation. Aggregates located farther from the root surface are more likely to exhibit delayed resource competition and community succession. Overcoming this limitation requires the integration of spatial tracing with microregional omics. Persistent luminescent and long-afterglow nanoplatforms have been used to localize submicrometer particles under low-autofluorescence backgrounds [20,21]. However, these approaches should be viewed as particle-localization tools rather than complete spatial-speciation methods. Their use should, therefore, be complemented by LA-ICP-MS, NanoSIMS, synchrotron-based µ-XRF/µ-XANES, STXM–NEXAFS, or Raman imaging to resolve elemental distribution, nanoscale particle–microbe associations, and chemical speciation. In future studies, coupling these platforms with LA-ICP-MS, metatranscriptomics, and targeted metabolomics could help test whether particle-enriched microregions correspond to microbial hotspots with enhanced As methylation, Fe cycling, or ligand secretion functions. Such spatial–functional alignment would advance nanomaterial microecology research from correlative inference toward more testable mechanistic models, although rigorous causal manipulation experiments remain necessary.

A critical limitation of the existing microbial evidence must be emphasized. The vast majority of studies cited in this section report changes in rhizosphere bacterial community composition based on 16S rRNA amplicon sequencing of bulk or homogenized rhizosphere soil. Such data demonstrate that ENM exposure is associated with shifts in relative taxonomic abundance, but they do not directly demonstrate that these shifts translate into altered biogeochemical functions. For example, the enrichment of taxa associated with Fe reduction or As methylation does not necessarily indicate increased Fe reduction rates or arsM expression in situ. Without parallel metatranscriptomic, proteomic, enzyme activity, stable-isotope probing, or targeted functional-gene expression data, the inference that “microbial community restructuring regulates Fe-mediated Cd/As fate” remains a plausible hypothesis rather than a demonstrated mechanism.

The microbial community responses described in Section 3.1, Section 3.2 and Section 3.3—ranging from toxic perturbation and adaptive reassembly to functional rewiring of Fe cycling, As transformation, and metabolite networks—do not operate independently of the interfacial chemistry described in Section 2. Rather, they are proposed to act as an intermediary regulatory layer that modifies the biogeochemical environment at each specific node of the contaminant migration pathway. Section 4 examines how these microbial functional changes are hypothesized to translate into alterations at four sequential nodes: rhizospheric front-end immobilization, root-surface iron-plaque gating, plant internal sequestration, and restricted long-distance transport. It is important to note, as emphasized in Section 3, that the mechanistic linkages between microbial community shifts and outcomes at these nodes are, in most cases, supported by correlative rather than directly causal evidence; they are presented as a working framework rather than an established mechanism.

## 4. Microbially Mediated Continuous Mechanistic Chain: From Front-End Immobilization to Restricted Cross-Interface Migration

**Figure 8:**
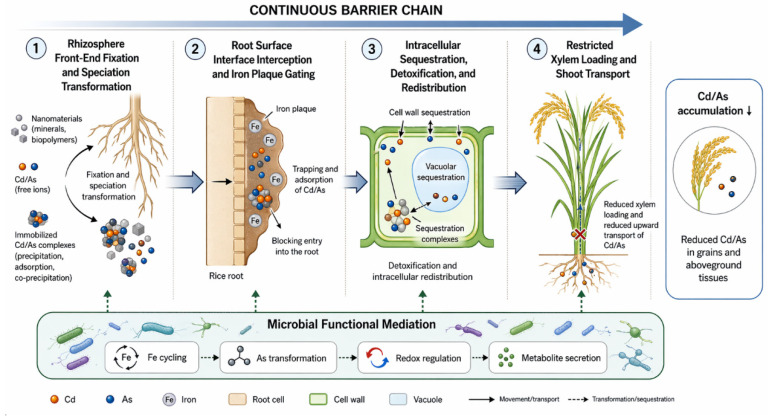
Microbially mediated continuous mechanistic chain: key nodes through which ENMs may regulate Cd/As accumulation in flooded rice. This conceptual synthesis integrates evidence from multiple studies and should not be interpreted as a single experimentally verified pathway. Solid arrows indicate relatively well-supported processes, whereas dashed arrows indicate proposed microbial mediation processes that remain partly correlative and require causal validation.

By integrating microinterface processes with functional responses, ENM-mediated regulation of Cd/As accumulation in flooded rice can be summarized as a continuous mechanistic chain consisting of rhizospheric front-end immobilization, root-surface iron-plaque gating, plant internal sequestration and detoxification, and restricted xylem transport (Figure 8). Within this chain, rhizosphere microbial networks are hypothesized to modulate contaminant-element migration fluxes and endpoint fate at each node by influencing redox processes, Fe cycling, and metabolite secretion. However, in most cases, this microbial contribution is inferred from community composition patterns, bulk elemental fractionation, or metabolite-related evidence rather than directly validated by functional assays or causal manipulation experiments. Importantly, the complete ENM transformation–microbial functional rewiring–Fe cycling–Cd/As flux–grain accumulation sequence has not yet been demonstrated within a single integrated experimental system.

### 4.1. Rhizospheric Front-End Immobilization and Reduced Bioavailability

The initial mitigation effects of ENMs occur primarily outside the root, with the key objective of reducing the mobility of bioavailable Cd/As. Biosynthesized Se NPs can decrease the proportion of acid-extractable Cd in soil and promote its transformation into the more stable oxidizable fraction [23]. In Cd/As co-contaminated systems, combined nZVI and melatonin intervention reduces soil-available Cd/As and is accompanied by coordinated changes in DCB-Fe, DCB-Cd, and DCB-As [24]. Studies on circulation-enhanced electrokinetic remediation of Cd/Pb-contaminated soils also indicate that heavy-metal migration and interfacial redistribution are controlled by coupled multi-process interactions rather than by immobilization alone [43]. Therefore, front-end immobilization is more appropriately understood as a reduction in bioavailability resulting from the combined effects of nanoscale adsorption, Fe-phase reconstruction, and potential microbially associated processes. Among these mechanisms, adsorption and Fe-phase reconstruction are relatively well-supported by physicochemical and fractionation evidence, whereas the specific contribution of microbial mediation remains less directly demonstrated and requires functional validation.

### 4.2. Root-Surface Iron-Plaque Interception and Regulation of Cross-Interface Flux

Cd/As reaching the root surface must undergo secondary screening at the iron-plaque interface. High-resolution elemental imaging shows that Cd, Se, and O colocalize in root-surface microregions under Se NP treatment, suggesting an evident interfacial retention of contaminant elements before they cross the cortex [23]. Dynamic accumulation of DCB-Cd and DCB-As fractions further indicates that root-surface iron plaque and adjacent mineral components are important physicochemical barriers limiting inward contaminant migration [24]. This gating effect is not constant. Based on correlative evidence from community composition studies, DCB fractionation, and rhizosphere chemistry, it has been proposed that reducing microbial communities, rhizosphere exudates, and organic ligands may collectively modulate the magnitude of interfacial retention. However, direct functional validation of the microbial contribution to iron-plaque gating under flooded rice conditions remains limited. Site competition, coordination complexation, and mineral-phase transformation may all modify the interfacial adsorption thermodynamics and ultimately affect the actual cross-interface influx.

### 4.3. Internal Sequestration and Detoxification Through Plant–Microbe Coordination

Cd/As that overcomes the root-surface barrier does not necessarily enter long-distance transport pathways. Instead, it may undergo local immobilization, complexation, or organellar sequestration. Under Cd stress, Se NPs can partially restore a disturbed endophytic community structure and activate glutathione metabolism, the phenylpropanoid pathway, and hormonal defense signaling [23]. These responses may promote the conversion of Cd into less toxic complexed forms and enhance its compartmentalization within vacuoles or cell walls. From this perspective, internal sequestration should not be viewed simply as a unidirectional plant stress response. Instead, it may reflect the combined influence of plant metabolic defense and microbially associated nutritional or signaling processes. Nevertheless, the extent to which microbial changes directly contribute to intracellular Cd/As complexation or compartmentalization remains unclear and should be tested using paired plant transcriptomics, endosphere microbiome profiling, and causal manipulation approaches.

### 4.4. Restricted Xylem Loading and Blocking of Grain Accumulation

The endpoint of the continuous mechanistic chain is reduced aboveground transport flux and decreased risk of grain accumulation. Front-end immobilization, iron-plaque retention, and internal sequestration collectively reduce the baseline load available for xylem loading. For example, combined nZVI and melatonin application significantly reduces Cd and As accumulation in grains without evident antagonism to Fe nutrition [24]. In Cd/As mitigation systems, regulatory strategies such as Se-Fe-P can reduce soil-available As, promote root-surface iron-plaque formation, and alter Fe speciation within iron plaque, thereby decreasing the risk of As migration to grains [40]. Therefore, improvements in the safety of agricultural products are more likely to result from the cumulative effects of external speciation transformation, root-surface retention, and internal compartmentalization. Microbial community changes may participate in these processes by modifying redox conditions, Fe cycling, and metabolite availability, but their causal contribution to reduced grain Cd/As accumulation remains to be quantitatively separated from direct ENM physicochemical effects.

Overall, the node-based chain proposed in this section should be interpreted as a working mechanistic framework rather than a fully validated causal pathway. The current evidence provides relatively strong support for several physicochemical nodes, including reduced contaminant bioavailability, iron-plaque-associated retention, and restricted grain accumulation. By contrast, the microbial mediation of these nodes is supported mainly by correlative evidence from community profiling, fractionation patterns, and metabolite-related observations. Future studies combining SynCom manipulation, metatranscriptomics, targeted metabolomics, and spatially resolved elemental imaging are required to determine whether microbial functional changes are causal drivers or secondary responses within ENM-mediated Cd/As mitigation systems.

To provide a more direct comparison of mitigation performance across material classes, we further summarized the reported percentage reductions in Cd and/or As bioavailability, uptake, translocation, or grain accumulation under different ENM treatments (Table 2). Because endpoints and experimental designs vary substantially among studies, the values should be interpreted as study-specific effect sizes rather than universally comparable efficiencies.

### 4.5. Theoretical Boundary Note: Sb as a Future Research Target

Antimony (Sb) is not addressed in this review as a validated target of ENM-based mitigation because direct evidence for Sb in nanomaterial–rice rhizosphere systems remains insufficient to support mechanistic interpretation. Its inclusion here is limited to a brief theoretical note: Sb(V) shares several coordination chemistry characteristics with As(V), including an affinity for Fe oxide surfaces and a sensitivity to Fe reduction–dissolution cycles, suggesting that aspects of the interfacial framework developed for As may serve as a starting conceptual template for future Sb research. However, this extrapolation is entirely theoretical. Validated evidence concerning microbial functional mediation, plant internal redistribution, or ENM effects on Sb speciation and mobility in rice systems is currently lacking, and Sb should not be treated as an equivalent case to Cd or As in the context of this review. We identify the establishment of direct field-relevant evidence for Sb in ENM–rice systems as a specific future research priority.

### 4.6. Relative Contributions of Physicochemical and Microbially Mediated Mechanisms: A Comparative Assessment

A balanced interpretation of ENM-mediated Cd/As mitigation in flooded rice systems requires distinguishing direct physicochemical processes from microbially mediated biogeochemical responses. Although this review emphasizes microbial mediation as a potential regulatory layer, the strongest evidence in many ENM-based remediation studies still supports physicochemical mechanisms, including adsorption, coprecipitation, Fe-phase reconstruction, mineral transformation, ion release, and redox-controlled speciation changes [44,45]. Therefore, microbial processes should not be interpreted as universally dominant; their contribution is likely to vary among the different nodes of the rhizosphere–iron plaque–plant continuum.

At the front-end immobilization node, direct geochemical immobilization is likely to be the primary driver in many systems. Evidence from changes in available Cd/As, sequential extraction fractions, DCB-extractable pools, and elemental colocalization more directly supports adsorption, precipitation, Fe-phase coupling, and contaminant partitioning than microbial causation [9,23,24]. At the iron-plaque gating interface, the relative contribution is more mixed: plaque formation and Cd/As retention are strongly controlled by radial oxygen loss, Fe oxide surface chemistry, sorption, and coprecipitation [3,4,17,25,46], but plaque stability and reactivity may also be modified by Fe-reducing or Fe-oxidizing microorganisms, root exudates, DOM, and low-molecular-weight organic acids [26,27,28,29]. For plant internal sequestration and restricted long-distance transport, the current evidence again favors plant physiological and physicochemical mechanisms, including cell-wall binding, vacuolar compartmentalization, phytochelatin complexation, transporter regulation, and xylem loading restriction [1,8,17,23,24,31]. Direct evidence that ENM-induced microbial functional changes causally regulate internal Cd/As sequestration or grain loading remains limited.

Overall, the current evidence supports a node-dependent interpretation: physicochemical mechanisms are most directly supported for front-end immobilization and plant internal sequestration, whereas microbial mediation is most likely to be functionally important at redox-sensitive interfaces, especially Fe cycling, iron-plaque stability, DOM-mediated ligand exchange, and As speciation transformation [3,4,17,25,26,27,28,29,46]. The relative importance of each mechanism likely depends on ENM type, soil redox status, organic matter composition, contaminant speciation, rice growth stage, and rhizosphere microbiome activity [44,45,47,48,49]. Future studies should explicitly partition these contributions using sterile or microbiome-manipulated controls, stable-isotope probing, metatranscriptomics, targeted functional-gene assays, and spatially resolved elemental/speciation imaging [20,21,38,50,51].

## 5. Boundary Constraints of Complex Environmental Scenarios on Microinterface Coupling Mechanisms

The mechanistic chain described above is derived mainly from relatively controlled experimental systems. In real paddy-field environments, agricultural management, global-change factors, and coexisting emerging contaminants can alter rhizosphere microinterface processes, thereby affecting not only the direction, magnitude, and applicability boundary of ENM-mediated mitigation effects but also their ecological safety and long-term sustainability. To convert these boundary conditions from a conceptual summary into a critical research framework, Table 3 summarizes the major perturbation nodes, mechanistic pathways, knowledge gaps, research priorities, and evidence levels associated with each complex scenario.

### 5.1. Compound Perturbations Associated with Elevated CO_2_ and Micro-/Nanoplastics

**Figure 9:**
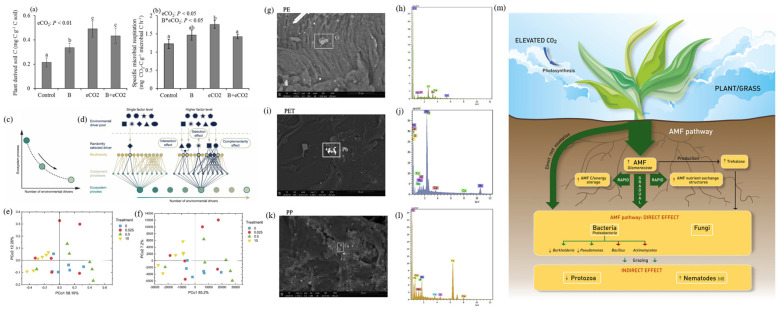
Composite figure showing rhizosphere carbon-flow regulation, microbial feedback, and contaminant carrier effects under eCO_2_ and micro-/nanoplastic coexposure. This composite figure integrates evidence from multiple independently published studies and does not represent a single experimentally validated system. Different panels are combined to illustrate potential boundary conditions that may modify ENM-mediated regulation in flooded rice rhizospheres. Notes: eCO_2_, elevated CO_2_; PGPR, plant growth-promoting rhizobacteria; AMF, arbuscular mycorrhizal fungi; MPs/NPs, microplastics/nanoplastics; Control: ambient CO_2_ without bacteria addition; B: ambient CO_2_ with bacteria addition; eCO_2_: elevated CO_2_ without bacteria addition; B + eCO_2_: elevated CO_2_ with bacteria addition. Error bars show the standard error of the mean (n = 6). The same letters denote non-significant differences between treatments (*p* > 0.05), whereas different letters indicate statistically significant differences between treatments (*p* < 0.05). (Panels (**a**,**b**) are reprinted from Ref. [53]. Panels (**c**,**d**) are reprinted with permission from Ref. [56]. Copyright 2019 American Association for the Advancement of Science. Panels (**e**,**f**) are reprinted with permission from Ref. [58]. Copyright 2018 Elsevier Ltd. Panels (**g**–**l**) are reprinted with permission from Ref. [55]. Copyright 2019 Elsevier Ltd. Panel (**m**) is reprinted with permission from Ref. [52]. Copyright 2010 National Academy of Sciences).

In the context of global change, elevated CO_2_ (eCO_2_) first alters plant carbon-allocation strategies and root exudation fluxes. eCO_2_ can enhance plant-derived carbon inputs and their allocation to soil microbial systems and can regulate microbial respiratory feedbacks and carbon-use efficiency through PGPR [53] (Figure 9a,b). In addition, eCO_2_ can drive the transfer of plant-fixed carbon via AMF to rhizosphere bacterial and fungal communities, thereby reshaping rhizosphere carbon-flow pathways and the associated microbial networks [52] (Figure 9m). Increased carbon input may alter DOM composition and further influence microbial metabolic networks and Fe reduction processes. Therefore, eCO_2_ may alter the adsorption and precipitation efficiency of ENMs at rhizosphere interfaces through a front-end “carbon-flow-microbe” perturbation.

Co-exposure to micro-/nanoplastics (MPs/NPs) may transform the effects of a single ENM into a compound perturbation process. The superposition of multiple global-change factors can directionally affect soil functions and microbial diversity, indicating that rhizospheric processes in real environments are often jointly driven by multiple factors [56] (Figure 9c,d). Nanoplastic exposure can induce differentiation of soil–animal gut microbial communities, suggesting that such particles may amplify the perturbation effects through food webs and microregional ecological networks [58] (Figure 9e,f). Meanwhile, microplastic surfaces can adsorb multiple metal elements and exhibit carrier effects [55] (Figure 9g–l). Accordingly, plastic particles may alter ENM retention at the root surface and contaminant-element migration through adsorption-site competition, contaminant carriage, and compound stress.

### 5.2. Fe/DOM Dynamics and Agricultural Management as Drivers

Fe valence cycling and DOM concentration fluctuations are key boundary conditions determining the strength of iron-plaque gating. When reductive redissolution dominates or when high concentrations of organic ligands, such as low-molecular-weight organic acids, are present, the complexation partitioning, hetero aggregation, and interfacial rerelease of ENMs and Cd/As may all change, thereby affecting the contaminant-element cross-interface influx. Water management, such as alternate wetting and drying (AWD), can also systematically reshape redox boundaries across soil profiles and the evolution of Fe mineral crystallinity [19]. In addition, the exposure pathway of ENMs, such as soil application or foliar application, determines their action nodes: the former is more likely to trigger rhizospheric front-end processes, whereas the latter may preferentially affect plant internal metabolic responses. These variables constitute constraints that must be considered when extrapolating the mechanistic chain across systems.

### 5.3. Nanotoxicity, Long-Term Persistence, and Ecological Risk Considerations in Paddy Systems

The preceding sections have mainly discussed the potential of ENMs to reduce Cd/As bioavailability, enhance root-surface retention, and restrict contaminant transport in flooded rice systems. However, a balanced assessment must also consider the potential adverse effects of ENM application, particularly because paddy soils are biologically active, redox-dynamic ecosystems, and because practical remediation may require repeated or large-scale field use.

Nanotoxicity is used to not target soil microorganisms. ENMs are not selectively active toward contaminant-mobilizing microorganisms. Their high surface reactivity, ion-release capacity, and potential to generate reactive oxygen species may also affect non-target microbial groups that are essential for soil fertility and ecosystem functioning [47,48,49]. For example, high-dose Ag NPs have been reported to suppress nitrogen-fixation activity and reduce the abundance of diazotrophic taxa, as discussed in Section 3.1 [39,61]. More broadly, metal-based ENMs may inhibit sensitive functional groups, including ammonia-oxidizing bacteria, nitrogen-fixing bacteria, methanotrophs, and mycorrhizal fungi [47,48,49,62]. Such effects could alter nitrogen cycling, phosphorus solubilization, methane oxidation, and organic matter decomposition [47,48,49]. Therefore, ENM-mediated mitigation of Cd/As accumulation should not be evaluated only by contaminant endpoints in rice tissues, but also by its consequences for microbial functional diversity and nutrient-cycling processes.

For the potential enhancement of metal mobility and unintended remobilization, although ENMs are often designed to immobilize Cd/As through adsorption, precipitation, or Fe-plaque-associated retention, their effects may not always be protective under complex field conditions. Changes in pH, DOM concentration, Fe redox cycling, and competing ions can alter ENM surface properties and may trigger the release of previously retained contaminants [27,28,29,44,45]. For example, high DOM concentrations or low-molecular-weight organic acids may promote the ligand-assisted mobilization of metals or metalloids [27,28,29,45], while the reductive dissolution of Fe phases may weaken iron-plaque retention and release associated As [17,41,46]. In addition, coexisting micro-/nanoplastics may compete with ENMs for adsorption sites or act as carriers for metal transport, as discussed in Section 5.1. Thus, ENMs may either decrease or, under certain boundary conditions, increase contaminant mobility, depending on the soil chemistry, water management, and coexisting colloidal particles.

For long-term persistence and bioaccumulation potential, unlike many organic amendments, most metal-based ENMs, such as Fe-based, Se-based, and CeO_2_ nanomaterials, do not simply degrade into innocuous products. Instead, they may undergo transformation, aggregation, dissolution, surface coating, or redistribution while remaining in the soil environment beyond a single rice-growing season [44,45,63,64]. The fate of these materials across repeated flooding–drainage cycles remains insufficiently characterized [45,64]. Their possible accumulation in root surfaces, soil aggregates, soil invertebrates, aquatic organisms in paddy water, or rice tissues also requires further investigation [20,21,45,63]. Persistent luminescence tracing, LA-ICP-MS imaging, synchrotron-based XRF/XANES, and isotope-labeling approaches could be combined in future multi-season studies to determine whether ENMs persist, transform, or enter food-web pathways under realistic paddy conditions.

As to the ecological risks from repeated field application, most existing studies are based on short-term pot or hydroponic experiments, whereas practical agricultural remediation may involve repeated ENM application over multiple seasons [23,24,32,40,64]. Under such scenarios, cumulative ENM loading may exceed the exposure levels tested in single-season experiments. Long-term accumulation could progressively select for tolerant microbial taxa while reducing sensitive but functionally important groups, potentially simplifying microbial networks and weakening ecosystem services such as carbon turnover, nitrogen transformation, organic matter decomposition, and methane regulation [48,49,61,64]. ENM particles or their transformation products may also be transported to adjacent aquatic ecosystems through irrigation return flow, runoff, or drainage, posing additional risks to aquatic microbial communities and biota [44,61,63].

Regarding field validation, scalability, economic feasibility, and regulatory considerations, a critical translational gap remains between the controlled-system evidence reviewed here and the requirements for practical field deployment. Most available studies are based on pot, hydroponic, or short-term microcosm experiments, whereas field-scale remediation must operate under heterogeneous soil chemistry, natural microbial communities, variable weather conditions, repeated flooding–drainage cycles, and agronomically realistic contamination levels. Therefore, the mitigation efficiency observed under controlled conditions should not be directly extrapolated to commercial rice-production systems without multi-season field validation.

Scalability also depends on material synthesis, formulation stability, delivery route, and compatibility with existing agronomic practices. ENMs that perform well at small experimental scales may be difficult to deploy over large paddy areas if they require complex synthesis procedures, high-purity precursors, repeated application, or specialized delivery equipment. Soil application may directly target the rhizosphere and iron-plaque processes but requires large material inputs, whereas foliar application may reduce soil loading but acts through different physiological pathways and may not fully address rhizospheric Cd/As bioavailability.

Economic feasibility is another major constraint. For ENM-based remediation to become practically relevant, its cost per unit area and per unit reduction in grain Cd/As should be evaluated against established amendments such as lime, biochar, silicon fertilizers, Fe-based soil amendments, organic amendments, and water-management strategies. Future studies should, therefore, report not only contaminant reduction efficiency, but also ENM dose, application frequency, synthesis route, crop-yield response, and estimated implementation cost.

From a regulatory perspective, current evidence remains insufficient to define safe application rates, acceptable residue thresholds, or long-term environmental fate criteria for ENM-based paddy remediation materials. Before large-scale application can be recommended, ENM remediation strategies should be evaluated using standardized risk-assessment frameworks that integrate contaminant mitigation efficiency with ecological safety endpoints, including microbial functional stability, nutrient cycling, greenhouse-gas-related processes, ENM persistence, off-site transport, and potential food chain transfer.

## 6. Methodological Frontiers: Spatial Tracing, Multi-Omics, and a Causal Evidence System

The analysis of ENM regulatory effects is shifting from endpoint correlative descriptions toward multi-node, verifiable mechanistic models. Achieving this transition requires an evidence system that simultaneously captures spatial distribution, functional expression, and causal manipulation (Table 4).

### 6.1. In Situ Characterization and High-Resolution Spatial Tracing: A Comparative Methodological Overview

Determining the actual spatial distribution and chemical speciation of ENMs and the associated contaminant elements within rhizosphere microinterfaces requires a toolkit of complementary high-resolution analytical methods, each offering distinct capabilities and limitations (Table 5).

Persistent luminescence and long-afterglow nanoplatforms offer the specific advantage of low-autofluorescence background imaging in complex plant and soil matrices, enabling particle tracking across the rhizosphere–iron plaque–cortex continuum without the signal interference inherent to conventional fluorescent labels [20,21]. Their primary limitation is that they are best suited as particle-localization tools; they do not provide elemental or chemical speciation information and currently require the introduction of exogenous tracer particles rather than directly labeling environmentally relevant ENMs.

Synchrotron-based micro-X-ray fluorescence (µ-XRF) and micro-X-ray absorption near-edge spectroscopy (µ-XANES) provide elemental mapping and valence-state speciation at a micrometer spatial resolution directly within hydrated or cryogenically preserved root cross-sections [4,38]. µ-XANES is particularly useful for determining As(III)/As(V) and Fe(II)/Fe(III) ratios at specific spatial locations within iron plaque, which is directly relevant to the gating function discussed in Section 2.2. Their primary limitation is the requirement for synchrotron beam access, which restricts throughput and routine application.

Laser ablation–inductively coupled plasma–mass spectrometry (LA-ICP-MS) enables spatially resolved multi-element quantitative mapping of root cross-sections and soil thin sections, typically at lateral resolutions of approximately 2–20 µm. It can simultaneously map ENM-derived elements, such as Fe, Se, or Ce, together with Cd and As, making it highly suited for evaluating colocalization and partitioning at the iron-plaque interface. Its limitation is that the analysis is destructive and does not directly provide chemical speciation information.

NanoSIMS provides nanoscale isotopic and elemental mapping, typically at tens to hundreds of nanometers in spatial resolution, making it valuable for resolving particle–microbe associations or element distributions at the single-cell and subcellular scales. However, its field of view is small, sample preparation is demanding, and chemical speciation information is limited. Its application to ENM–rice rhizosphere systems remains underdeveloped and represents an important opportunity for future work.

Scanning transmission X-ray microscopy combined with near-edge X-ray absorption fine structure spectroscopy (STXM–NEXAFS) provides nanoscale to submicrometer chemical imaging and can help bridge the gap between elemental mapping and chemical speciation. It is particularly useful for mapping organic functional groups, mineral-associated carbon, and selected metal redox states in thin hydrated or cryo-preserved samples. However, it requires specialized synchrotron facilities and thin-section preparation.

Confocal Raman imaging provides non-destructive, label-free chemical identification of mineral phases and organic functional groups at micrometer-scale resolution. It may help identify ENM transformation products, organic coatings, and mineral phases within rhizosphere aggregates or iron-plaque regions. Its main limitations are relatively weak signals, fluorescence interference in organic-rich matrices, and limited sensitivity for trace metals compared with mass spectrometry or synchrotron-based methods.

Overall, no single spatial method can simultaneously resolve particle localization, elemental distribution, chemical speciation, and microbial function. A multimodal workflow combining luminescence tracing for particle localization, LA-ICP-MS or µ-XRF for quantitative elemental mapping, µ-XANES or STXM–NEXAFS for speciation, NanoSIMS for nanoscale particle–microbe associations, and Raman imaging for mineral/organic phase identification would provide a more balanced evidence base for validating ENM behavior across rhizosphere–iron plaque–plant tissue interfaces. Coupling these spatial methods with microregional metatranscriptomics or laser-capture microdissection followed by RNA sequencing would further connect particle distribution with local microbial functional expression.

### 6.2. Integrated Meta-Omics and Deconstruction of Causal Networks

To demonstrate that “community shifts” represent “functional rewiring,” study designs should extend from bulk amplicon sequencing to functional and causal validation. First, metatranscriptomics and targeted RT-qPCR should be used to determine whether genes involved in As methylation, Fe oxidation/reduction, sulfur metabolism, siderophore biosynthesis, and organic acid secretion are actively expressed under ENM exposure. Priority targets include arsM and other As-transformation genes, Fe(II)-oxidation and Fe(III)-reduction gene clusters, sulfate-reduction genes, and the genes involved in siderophore production.

Second, metaproteomics and enzyme-activity assays are needed to test whether transcript-level changes translate into active biochemical processes. This is particularly important because changes in gene abundance or transcript abundance do not necessarily correspond to realized rates of Fe reduction, As methylation, or ligand-mediated mobilization. Third, stable-isotope probing using labeled substrates, such as ^13^C-labeled root exudate analogs, ^13^C-labeled organic acids, or ^34^S-labeled sulfate, could help identify the active microbial populations responsible for carbon transformation, ligand production, or sulfur-mediated Fe–As coupling.

Finally, synthetic microbial communities (SynComs), gnotobiotic rhizosphere cultivation, and pathway-specific inhibition should be integrated with spatially resolved elemental imaging to establish causal attribution. For example, comparing ENM-treated rice systems inoculated with complete SynComs, Fe-cycling-deficient SynComs, or arsM-deficient microbial assemblages would help distinguish whether observed reductions in Cd/As accumulation are driven primarily by direct ENM physicochemical retention, microbial functional activity, or plant physiological responses. Such a workflow would move the field from compositional correlation toward experimentally testable causal networks. 

## 7. Conclusions and Perspectives

This review starts from the microinterface of the flooded rice rhizosphere and integrates the environmental behavior, microbial responses, and plant internal processes involved in the ENM-mediated regulation of Cd/As accumulation. The current evidence, synthesized from independent studies across multiple experimental systems, supports the view that ENM effects are not limited to direct adsorption or precipitation, but may jointly influence contaminant-element fate through continuous nodes, including rhizospheric front-end immobilization, root-surface iron-plaque gating, microbial functional regulation, plant internal compartmentalization, and restricted long-distance transport. The potential coupling between microinterface material partitioning and rhizosphere microbial metabolic expression, such as Fe cycling, As transformation, and ligand secretion, represents an important entry point for understanding the biogeochemical fate of Cd/As. However, the causal contribution of microbial functional changes must be separated from direct ENM physicochemical effects through coordinated spatial imaging, functional omics, and microbial manipulation experiments.

Based on the critical gaps identified in this review, we propose the following five specific research priorities for the field:

(1) Identification and functional characterization of the key microbial taxa regulating Fe-mediated Cd/As transformations under ENM exposure: The current evidence is still dominated by compositional 16S rRNA surveys and co-occurrence analyses. Future work should apply metatranscriptomics, stable-isotope probing, and SynCom-based manipulation experiments to identify which specific microbial taxa and functional guilds are causally responsible for the biogeochemical changes attributed to microbial mediation. Priority should be given to rice-relevant Fe-reducing bacteria, As-methylating microorganisms, siderophore producers, sulfur-cycling taxa, and *arsM*-carrying microbial lineages;
(2) Development of standardized protocols for ENM exposure studies in flooded rice systems: Quantitative comparison across studies remains difficult because of the variation in ENM synthesis routes, particle characterization, soil type, contamination level, rice cultivar, exposure route, and experimental duration. Future studies should adopt minimum reporting standards for ENM physicochemical properties, including size distribution, surface area, zeta potential, aggregation state, dissolution rate, and transformation products, together with soil redox status, Fe/DOM background, water-management regime, contamination level, and exposure duration;
(3) Integrated multimodal spatial omics for mechanistic validation: Mechanistic validation of the proposed framework requires spatial alignment of particle localization, elemental distribution, Fe speciation, and microbial functional expression within the same rhizosphere microdomains. Complementary spatial methods, including luminescence tracing, LA-ICP-MS, NanoSIMS, synchrotron-based µ-XRF/µ-XANES, STXM–NEXAFS, and Raman imaging, should be integrated with metatranscriptomics, targeted metabolomics, and laser-capture microdissection RNA sequencing to establish a closed evidence chain linking spatial localization, functional expression, and endpoint Cd/As accumulation;
(4) Long-term multi-season field monitoring of ENM fate, efficacy, and ecosystem effects: Long-term safety assessment under complex field scenarios should become a core component of ENM-based paddy remediation research [44,45,49,61,63,64]. Future studies should move beyond short-term pot experiments and systematically track ENM transformation, persistence, off-site transport, Cd/As bioavailability, grain accumulation, rice yield, microbial functional stability, nutrient cycling, and greenhouse-gas-related processes under realistic agricultural scenarios involving AWD water management, repeated field application, micro-/nanoplastic coexistence, and extreme climatic events;
(5) Safe-by-design ENM development integrating efficacy, ecological compatibility, and regulatory feasibility: Future ENM design for agricultural remediation should incorporate environmental safety as a core design criterion alongside contaminant mitigation efficiency [45,48,49,69]. This includes developing ENMs with predictable transformation pathways, minimized non-target toxicity to soil functional guilds, reduced long-term persistence risk, and economically feasible synthesis and application routes. Collaboration among materials scientists, soil microbiologists, agronomists, toxicologists, and regulatory scientists will be essential for translating mechanistic insights into safe, scalable, and field-relevant remediation technologies.

## Figures and Tables

**Table 1:** Major action nodes, microbial responses, and evidence characteristics of different ENMs at microinterfaces of the flooded rice rhizosphere.

ENM Type	Representative Materials	Primary Action Nodes and Front-End Behavior	Microbial Ecological/Functional Response	Effects on Cd/As	Evi-Dence Level *	References
Fe-based ENMs	nZVI, Fe-MOFs, etc.	Front-end immobilization; iron-plaque gating	Functional remodeling related to Fe cycling	Reduced bioavailable Cd/As and promoted root-surface retention	+++	[3,4,9,24]
Se-based ENMs	Se NPs	Front-end reconstruction; internal response	Enrichment of beneficial bacteria; activation of detoxification metabolism	Reduced Cd/As bioavailability and enhanced internal sequestration	+++	[23,31]
Rare-earth oxides	CeO_2_ NPs	Front-end transformation; interfacial partitioning	Metabolite-driven surface reduction	Altered particle interfacial reactivity	++	[22,32]
Carbon-based functional materials	Carbon dots, carbon nanosol, etc.	Front-end immobilization; functional mediation	PGPR enrichment; network rewiring	Stress alleviation and restricted long-distance transport	++	[33,34,35,36]
Persistent luminescent ENMs	Long-afterglow/persistent luminescent particles	Spatial validation	Particle-localization anchors	Used for mechanistic validation rather than direct mitigation	+	[20,21,37,38]

* Evidence level criteria: Evidence levels (+, ++, +++) were assigned based on four dimensions evaluated collectively: (i) directness, whether the cited evidence directly demonstrates the stated process in rice or flooded paddy systems; (ii) methodological quality, whether spatially resolved, functional, or mechanistic approaches were used rather than only bulk extraction or amplicon-based methods; (iii) consistency, whether similar findings have been reported across independent studies, materials, or systems; and (iv) causal support, whether the proposed mechanism has been supported by experimental manipulation rather than only correlative observations. +++ indicates relatively strong evidence with direct, methodologically robust, and consistent support; ++ indicates moderate evidence based on direct or semi-direct observations but limited causal validation; + indicates preliminary, indirect, methodological, or conceptual evidence. These ratings are intended to guide interpretation of the current evidence base and do not constitute a formal systematic review grading scheme.

**Table 2:** Reported percentage reductions in Cd and As bioavailability, uptake, translocation, or accumulation under different ENM treatments.

ENM Category	Representative Material	System	Endpoint	Cd Reduction (%)	As Reduction (%)	References
Fe-based ENMs	nZVI + melatonin	Pot, flooded rice	Grain Cd/As	>85	>85	[24]
Fe-based ENMs	Fe-MOFs, foliar application	Paddy rice	Grain Cd/inorganic As	67.8	22.8	[9]
Se-based ENMs	Se NPs	Pot, flooded rice	Cd fractionation/uptake	NR	N/A	[23]
Rare-earth oxide ENMs	CeO_2_ NPs	Rice-planted soil/model rhizosphere	ENM transformation	NR	NR	[22,32]
Carbon-based materials	Carbon dots/carbon nanosol	Rice or related crop system	Cd/As uptake or stress response	NR or reported value	NR	[33,34,35,36]
Mixed ENMs	Multiple types	Meta-analysis	Pooled metal(loid) accumulation	NR	NR	[10]

Note: NR, not reported or not directly calculable from the cited study. Percentage reductions were included only when explicitly reported or directly calculable. CeO_2_ NPs and carbon-based ENMs were retained for mechanistic evidence but not for quantitative Cd/As reduction. Mixed ENMs report overall metal(loid) accumulation reduction only, not species-specific Cd/As values.

**Table 3:** Boundary conditions and environmental modifiers affecting ENM-mediated Cd/As mitigation in flooded rice rhizospheres.

Complex Scenario	Primary Perturbation Node	Core Mechanistic Pathway	Potential Effect on the Continuous Mechanistic Chain	Key Knowledge Gaps	Specific Research Priorities	Evidence Level	References
eCO_2_	Rhizosphere carbon-flow and microbial networks	Altered root exudation and DOM composition, stimulating Fe reduction and microbial metabolic activity	Rewrites the front-end immobilization baseline and microbial mediation layer	How does eCO_2_ specifically modify ENM surface chemistry and partitioning?	FACE experiments integrating ENM application, DOM profiling, and Fe speciation under eCO_2_	++, indirect; no direct eCO_2_ × ENM rice evidence	[52,53,54]
Micro-/nanoplastic coexposure	Front-end interfacial competition and community stress	Adsorption-site competition, carrier effects, compound stress responses, and physical disturbance of the mucilage layer	Weakens individual ENM immobilization and reshapes root-surface cross-interface flux	Competitive adsorption kinetics between MPs/NPs and ENMs for iron-plaque binding sites remain unquantified	Competitive adsorption isotherms and realistic MP/NP coexposure pot experiments	++, carrier effects shown; direct ENM competition limited	[55,56,57,58,59,60]
Fe status and DOM fluctuations	Root-surface gating and interfacial partitioning	Iron-plaque formation/dissolution, coordination complexation, and adsorption thermodynamics	Amplifies or reverses iron-plaque gating strength	Threshold Fe/DOM ratios controlling retention-to-release transitions are unknown	Time-series DCB-Fe, porewater Fe^2+^, DOM, and Cd/As monitoring under ENM amendment	+++, strong mechanistic basis; thresholds unclear	[3,4,24,27,29]
AWDwater management	Redox boundaries and microecological succession	Eh fluctuations, metalloid reductive release, and Cd stabilization thresholds	Determines whether the first half of the mechanistic chain can be stably initiated	How ENMs behave under repeated flooding–drying cycles remains insufficiently tested	Long-term AWD experiments with ENM fate, Cd/As flux, and microbiome monitoring	++, water-management effects known; ENM-specific evidence limited	[2,8,19]

Note: * eCO_2_, elevated CO_2_; MPs/NPs, microplastics/nanoplastics; DOM, dissolved organic matter; AWD, alternate wetting and drying irrigation; FACE, free-air CO_2_ enrichment. Evidence levels indicate the strength of support for each boundary condition in the context of ENM-mediated Cd/As mitigation in flooded rice rhizospheres: +++, relatively strong mechanistic basis but still requiring quantitative threshold validation; ++, indirect or scenario-specific evidence with limited direct ENM–rice validation.

**Table 4:** Evidence categories, validation methods, and key limitations for major nodes in the proposed ENM-mediated microinterface mechanism chain.

Node	Question	Evidence (Cd/As) *	Evidence Category	Validation Methods	Main Limitation	References
Front-end immobilization	Is bioavailable Cd/As reduced before root entry?	+++/++	Category A/B: relatively direct evidence supports reduced bioavailable Cd/As, but the contribution of microbial mediation is often inferred from correlative evidence.	Sequential fractionation; rhizosphere metabolomics; microregional X-ray absorption spectroscopy	Lack of synchronous in situ evidence for particles, Fe phases, and elements	[8,23,24,27,29]
Plaque gating	Are Cd/As or ENMs retained at the iron-plaque/root-surface interface?	+++/+++	Category A: direct evidence from rice or closely related systems supports root-surface retention; Category B for the specific microbial contribution to plaque dynamics.	Luminescence tracing; LA-ICP-MS imaging; μ-XRF	Insufficient quantification from outer plaque to cortex	[3,4,20,23,24,25]
Microbial mediation	Do microbial communities causally drive elemental transformation?	+/++	Category B/C: mainly supported by community composition, metabolite profiles, and bulk fractionation; direct causal evidence remains limited.	Metatranscriptomics; targeted metabolomics; SynComs; gnotobiotic validation	Lack of causal linkage between functional expression and endpoints	[22,32,33,34,39,42]
Internal sequestration	Is long-distance transport restricted through internal sequestration or reduced xylem loading?	++/++	Category B: supported by tissue-level, subcellular, and plant-response evidence, but separation from front-end interception remains incomplete.	Subcellular elemental imaging; plant transcriptomics; spatial quantitative mass spectrometry	Difficult separation of front-end interception from downstream compartmentalization	[1,8,23,24,31]

* Evidence levels are indicated separately for Cd/As. +++ indicates experimentally demonstrated evidence in rice or closely related paddy systems with relatively direct mechanistic support (Category A); ++ indicates correlative support based on community composition, bulk elemental fractionation, metabolite profiling, or plant-response evidence without direct functional validation in the flooded rice rhizosphere (Category B); + indicates conceptual extrapolation from model systems or theoretical prediction requiring future experimental validation (Category C). SynComs, synthetic microbial communities; LA-ICP-MS, laser ablation–inductively coupled plasma–mass spectrometry; μ-XRF, micro-X-ray fluorescence imaging; μ-XANES, micro-X-ray absorption near-edge structure spectroscopy.

**Table 5:** Comparative spatial methods for resolving ENM behavior and contaminant fate at flooded rice rhizosphere microinterfaces.

Method	Main Information Provided	Approximate Spatial Resolution	Strength in ENM–Rhizosphere Studies	Main Limitation	References
Persistent luminescence/long-afterglow tracing	Particle localization and transport path	µm-scale imaging	Low background interference in plant tissues; useful for tracking particle movement	Does not provide elemental speciation; requires tracer particles	[20,21]
µ-XRF	Spatial elemental mapping	µm scale	Maps ENM-derived elements with Cd/As/Fe at root and iron-plaque interfaces	Limited speciation information without XANES	[4,31]
µ-XANES	Elemental valence/speciation	µm scale	Determines As, Fe, or Ce redox states at selected microregions	Requires synchrotron access; low throughput	[4,15,31]
LA-ICP-MS	Quantitative multi-element imaging	~2–20 µm	Quantifies co-localization of ENM-derived elements, Cd, As, and Fe	Destructive; no direct speciation	[65,66]
NanoSIMS	Isotopic and elemental nanoscale mapping	~50–100 nm	Resolves particle–cell or particle–microbe associations	Small field of view; demanding preparation; limited speciation	[67]
STXM–NEXAFS	Chemical-state and organic/mineral phase mapping	~20–50 nm	Links nanoscale chemical speciation with mineral/organic interfaces	Requires thin samples and synchrotron access	[66]
Confocal Raman imaging	Label-free mineral/organic phase identification	µm scale	Identifies mineral phases, organic coatings, and transformation products	Fluorescence interference; limited trace-metal sensitivity	[68]

Note: These methods are complementary rather than interchangeable. Combining particle-localization tools, quantitative elemental imaging, and chemical-speciation methods is necessary to establish a robust spatial evidence chain linking ENM localization, Fe-plaque transformation, Cd/As partitioning, and microbial functional hotspots.

## Data Availability

No new data were generated or analyzed in this study. This article is a review of previously published research; all data discussed are available in the original publications cited in the reference list.
